# Early warnings and repayment plans: novel trial management methods for monitoring and managing data return rates in a multi-centre phase III randomised controlled trial with paper Case Report Forms

**DOI:** 10.1186/s13063-019-3343-2

**Published:** 2019-04-27

**Authors:** William J. Cragg, Fay Cafferty, Carlos Diaz-Montana, Elizabeth C. James, Johnathan Joffe, Monica Mascarenhas, Victoria Yorke-Edwards

**Affiliations:** 1 MRC Clinical Trials Unit at UCL, London, UK; 2 Clinical Trials Research Unit, Leeds Institute of Clinical Trials Research, University of Leeds, Leeds, LS2 9JT UK; 3 Calderdale & Huddersfield NHS Foundation Trust, Huddersfield, UK

**Keywords:** Data return rates, Case Report Form returns, Data completeness, Trial management, Data management, Central monitoring

## Abstract

**Background:**

Monitoring and managing data returns in multi-centre randomised controlled trials is an important aspect of trial management. Maintaining consistently high data return rates has various benefits for trials, including enhancing oversight, improving reliability of central monitoring techniques and helping prepare for database lock and trial analyses. Despite this, there is little evidence to support best practice, and current standard methods may not be optimal.

**Methods:**

We report novel methods from the Trial of Imaging and Schedule in Seminoma Testis (TRISST), a UK-based, multi-centre, phase III trial using paper Case Report Forms to collect data over a 6-year follow-up period for 669 patients. Using an automated database report which summarises the data return rate overall and per centre, we developed a Microsoft Excel-based tool to allow observation of per-centre trends in data return rate over time. The tool allowed us to distinguish between forms that can and cannot be completed retrospectively, to inform understanding of issues at individual centres. We reviewed these statistics at regular trials unit team meetings. We notified centres whose data return rate appeared to be falling, even if they had not yet crossed the pre-defined acceptability threshold of an 80% data return rate. We developed a set method for agreeing targets for gradual improvement with centres having persistent data return problems. We formalised a detailed escalation policy to manage centres who failed to meet agreed targets. We conducted a post-hoc, descriptive analysis of the effectiveness of the new processes.

**Results:**

The new processes were used from April 2015 to September 2016. By May 2016, data return rates were higher than they had been at any time previously, and there were no centres with return rates below 80%, which had never been the case before. In total, 10 centres out of 35 were contacted regarding falling data return rates. Six out of these 10 showed improved rates within 6–8 weeks, and the remainder within 4 months.

**Conclusions:**

Our results constitute preliminary effectiveness evidence for novel methods in monitoring and managing data return rates in randomised controlled trials. We encourage other researchers to work on generating better evidence-based methods in this area, whether through more robust evaluation of our methods or of others.

## Background

Complete and timely reporting of trial data from investigator to sponsor is a key process in Good Clinical Practice in clinical trials [1]. There are various reasons why maintaining a complete dataset on an ongoing basis is important in trial management. An unreasonable delay between trial assessments or events at centres and data being available in trial systems means reduced oversight for the sponsor or clinical trials unit (CTU), and an impaired ability to monitor the trial in line with the expectations of Good Clinical Practice—that is, to ensure the trial is ‘… conducted, recorded and reported in accordance with the protocol, Standard Operating Procedures … Good Clinical Practice … and the applicable regulatory requirements’ [1]. This is particularly problematic for trials relying more on central than on-site monitoring, as many academic-led trials do [2]. Oversight committees’ decision-making may be impaired by reviewing trial data that are not complete. Trials with adaptive designs, in particular, need complete data for interim analyses to support robust decision-making about matters such as stopping recruitment to comparisons in multi-arm, multi-stage trials [3]. The CTU trial team’s ability to spot patient safety or protocol adherence problems in a timely manner is also reduced by delays in data returns. In the current climate regarding clinical trial monitoring, ‘risk-based monitoring’, supported by various regulators [4–6], often implies reduced reliance on on-site monitoring and increased use of central monitoring techniques. The usefulness of such techniques is largely dependent on having complete data at any given time. Maintaining a complete dataset is helpful in preparing for planned interim and final analyses, reducing the need for intense data chasing and cleaning work prior to database lock. Data backlogs have to be dealt with before final trial analyses, so may delay the release of trial results in some cases [7]. Finally, it is possible that data reported sooner are of higher quality, or at least that earlier submission allows sponsors to highlight issues sooner. For practical reasons, centres may also be able to respond more easily to data queries closer to the time of assessment.

Data management processes were highlighted in a recently published review of sources of inefficiencies in UK CTUs [7]. There is very little published evidence about the best methods for maintaining complete data throughout the lifetime of a trial, despite the importance of doing so. There is some evidence that electronic data capture systems may reduce the time to data availability in trial databases [8, 9] and that they can be used to direct data submission reminders to participating centres [10]. However, whether these advantages lead to consistently more complete data is not proven and, in any case, electronic data capture may not yet have fully replaced paper-based methods [11]. Others have helpfully reported their methods for reporting on data returns [12–15], but have not explained how they can be used to ensure consistently complete data in a trial. An exercise to identify standard requirements for data management systems in clinical trials recommends mechanisms to identify and report on missing or late data, but does not mention maintenance of high data returns throughout a trial [16]. Two recent papers offering advice on data management plan development also do not provide guidance on this issue [17, 18].

In our experience, a common method for maintaining complete data involves distributing lists of all currently overdue forms to participating centres at regular intervals, and requesting that centres return all of them within a set timeline. Centres requiring additional attention can be identified by use of acceptability thresholds, or ‘traffic light’ systems (i.e. thresholds used to assign acceptable centres a green label, at-risk centres amber and problem centres red). However, these are usually based on data from one point in time and do not easily show us if a centre is falling from green to amber to red. Equally, unless we are closely observing all centres in the red, we cannot easily see if they might actually be improving, and might therefore benefit from reward and further encouragement. Some trialists report using low data returns as a ‘trigger’ for on-site monitoring visits [19, 20], but there is no good evidence that additional visits to struggling centres improve data returns in the short or long term. In any case, the rationale is not clear: if a common cause of low data returns was under-resourcing at a centre, the loss of another day to monitoring activity will not help.

Robust, evidence-based methods to ensure consistently complete data would support oversight of trials, including central monitoring processes used within a risk-based monitoring framework, and may make trials more efficient overall in reducing delays in obtaining final results. From our experience, a small number of centres will have persistently low data returns for long periods during a trial, indicating that current practices in this area may not be optimal. In this article, we describe novel methods for identifying problems early and for managing problems when they arise, and we present some preliminary evidence for the effectiveness of these methods from a multi-centre, secondary care trial using paper Case Report Forms (CRFs).

## Methods

### Setting

The Trial of Imaging and Schedule in Seminoma Testis (TRISST; ClinicalTrials.gov, NCT00589537) is a phase III trial with a non-inferiority, factorial design, aiming to evaluate whether men who have had surgery for early stage testicular cancer and who are undergoing active surveillance can avoid unnecessary radiation exposure by reducing the number of computed tomography (CT) surveillance scans or by replacing standard CT scans with MRIs [21].

The trial recruited 669 participants between 2007 and 2014 from 35 UK centres, and will continue follow-up until 2020, reflecting the relatively good prognosis in these patients. The primary outcome is relapse with advanced disease. Secondary outcomes include disease-free and overall survival, and health economic and quality of life outcomes. TRISST is sponsored by the Medical Research Council, funded by Cancer Research UK, and run through the Medical Research Council Clinical Trials Unit at University College London (MRC CTU at UCL).

**Fig. 1:**
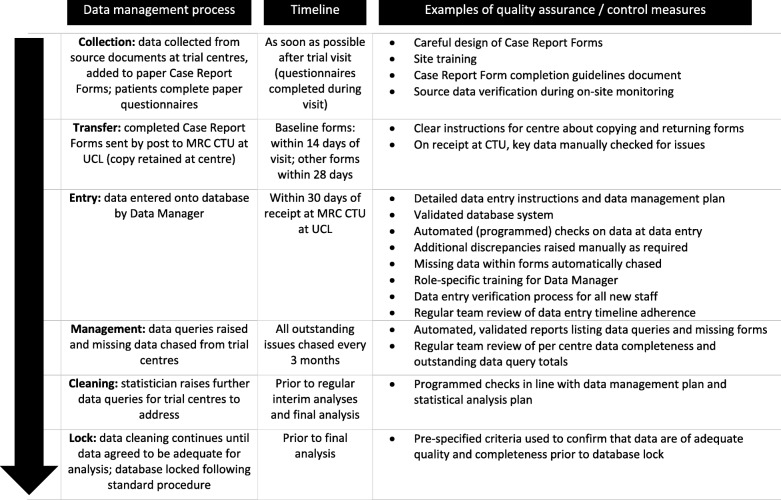
Summary of general processes for data collection, data cleaning and data quality assurance in TRISST. Note that the detail in this figure is presented to give context of data management in TRISST. The principle focus of the current work is in the ‘Management’ row, specifically how to most usefully review and act on data about trial data completeness. MRC CTU at UCL Medical Research Council Clinical Trials Unit at University College London, TRISST Trial of Imaging and Schedule in Seminoma Testis

**Fig. 2:**
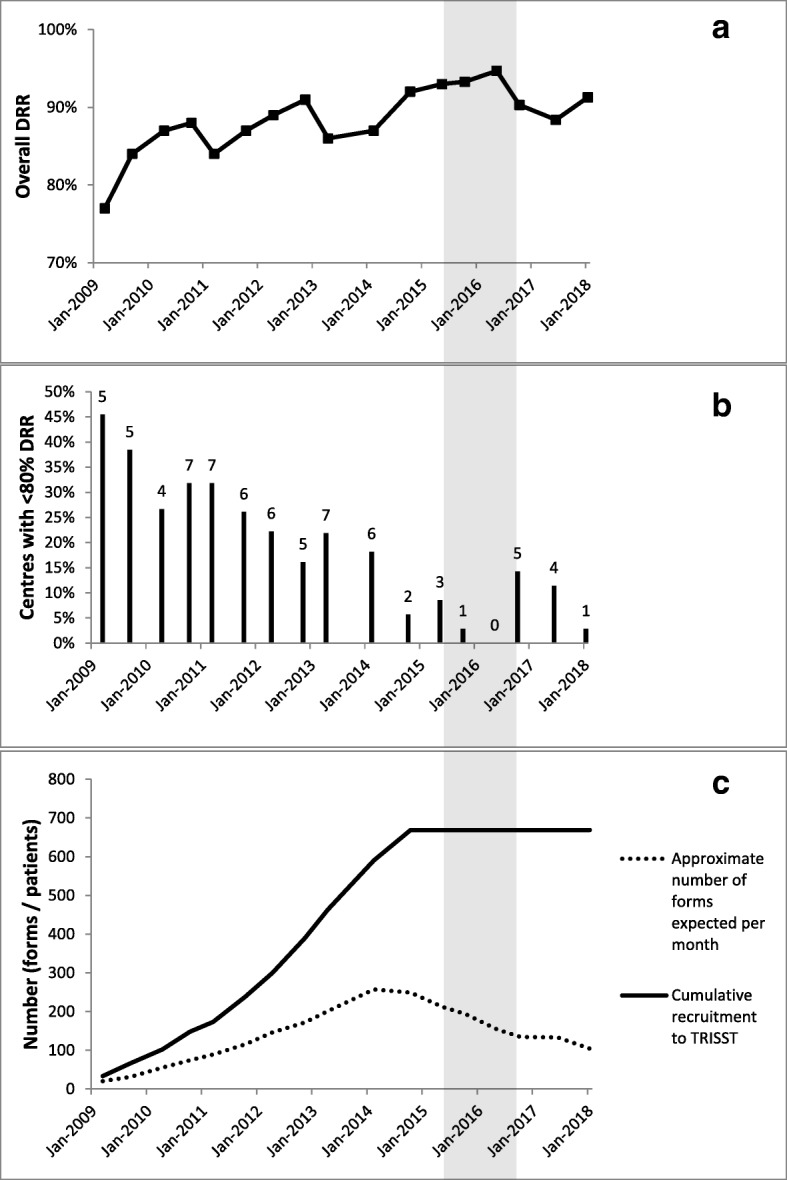
Changes in data return rates over the course of TRISST. **a** Overall data return rate (DRR) as reported at each Trial Management Group meeting. **b** Proportion of all centres with <80% overall DRR (number of centres given above each column); note that the reduction in proportion in the first years of the trial was mainly due to increasing numbers of centres participating in the trial. **c** Overall trial recruitment and per-month number of Case Report Forms expected, for context. Shaded area shows the time when the new methods, described in this article, were used. TRISST Trial of Imaging and Schedule in Seminoma Testis

Figure 1 shows the data collection and management processes for TRISST, and the various quality control and assurance processes in place, including a data management plan. The trial has relatively low data collection demands, perhaps due to not involving an Investigational Medicinal Product. Data entry and query management have been handled by, at most, one full-time equivalent data manager throughout the trial to date. Data are collected on paper CRFs, posted to the CTU (with a copy retained at the centre) and entered into a data management system (Elsevier’s MACRO [22]) by CTU staff. At randomisation, centres provided two pages of CRFs for each patient, and a two-page patient-reported outcome questionnaire (a modified EQ-5D [23]). Follow-up visits require another two pages of CRFs, with an additional EQ-5D questionnaire at some visits. Follow-up visits are largely aligned with standard practice (although this can vary between centres): every 3 months for 2 years, then every 4 months for the third year and then every 6 months up to 6 years (therefore 17 visits in total). Additional forms are required for specific events such as relapse or equivocal scan results. For a patient reaching the end of the follow-up schedule without any such unscheduled forms (as many patients do), we would expect to receive 52 pages of CRFs, with 16 of these being patient completed. Over the course of the trial, as many as 18,000 CRFs will be collected. Figure 2 shows the variation in the number of CRFs expected per month during the course of the trial.

Table 1 presents the terminology and ‘form statuses’ used for managing data returns in TRISST. At the start of 2015 (over 6 months after the end of recruitment), the overall data return rate (DRR) in TRISST was around 92%, and at most Trial Management Group (TMG) and CTU team reviews the DRR had been between 85 and 90% since near the start of recruitment (median across all TMG reviews: 87%). The number of CRFs expected per month at this time was around 200–250. The CTU team had used a threshold of 80% (based on experiences in other trials at MRC CTU at UCL) to indicate which centres might require attention and support to improve data returns during the conduct phase of the trial. This would also help with the aim to achieve 100% data returns by the time of database lock. TMG meetings had taken place approximately twice a year since the start of the trial, and each meeting report had given DRR figures overall, by CRF type and by centre. The median number of centres whose data returns were beneath the threshold in each TMG report was six. There were some persistent problem centres: four had < 80% DRR in over half of the TMG meeting reports. Until 2015, the process for dealing with centres with a DRR beneath 80% had been less formalised, but in general they had been contacted with a list of all outstanding CRFs and a request for outstanding data to be sent in, and to notify the CTU team of any current barriers to data returns.

**Table 1:** key terminology and calculations

Term^a^	Definition
Case Report Form due date	Date of the event that a form is linked to. For example, for a 6-month post-randomisation follow-up visit, a follow-up form due date would be the date of randomisation plus 6 months
Tolerance period	Short period after the due date (e.g. 14 or 28 days) for the participating centre to complete and return the relevant Case Report Forms. Forms are not considered overdue until this period has passed
Scheduled	Form due date is in the future
Expected	Form due date has passed, but the tolerance period has not. For the purposes of calculating the data return rate, these are counted as not due yet (the same as Scheduled forms)
Received	Form has been received and entered into the trial database system
Overdue	The tolerance period has passed, but the form has not yet been received at the clinical trials unit
Unobtainable	Participating centre has confirmed that data are not available and no form will be sent^b^
Data return rate (overall)	$\frac{Received}{Received+ Overdue+ Unobtainable}$
Date return rate (excluding unobtainable forms)^c^	$\frac{Received}{Received+ Overdue}$

^a^Form statuses (Scheduled, Expected, Received, Overdue, Unobtainable) are mutually exclusive categories

^b^Forms are designated Unobtainable to mean no further requests for the data will be made. These forms can either be included in data return rate statistics (as permanently missing) or excluded

^c^Excluding Unobtainable forms can be useful for looking only at unresolved problems, and excluding historical issues that cannot be resolved retrospectively such as missed patient-reported outcomes

As data completeness was identified by the CTU team as a priority for the trial’s follow-up phase (particularly for primary outcome data about late relapses, i.e. occurring after 36 months of follow-up), we decided to develop a more comprehensive process for handling the DRR than had been used previously.

### Data return rate reporting

**Fig. 3:**
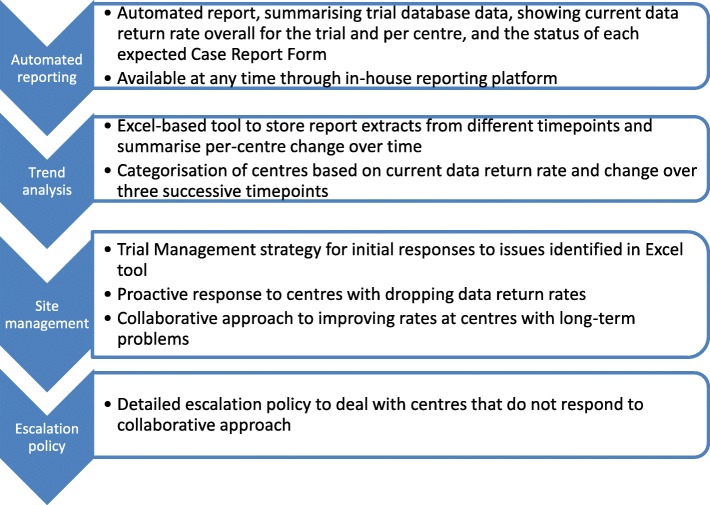
Summary of TRISST data return rate monitoring methods and supporting systems

**Fig. 4:**
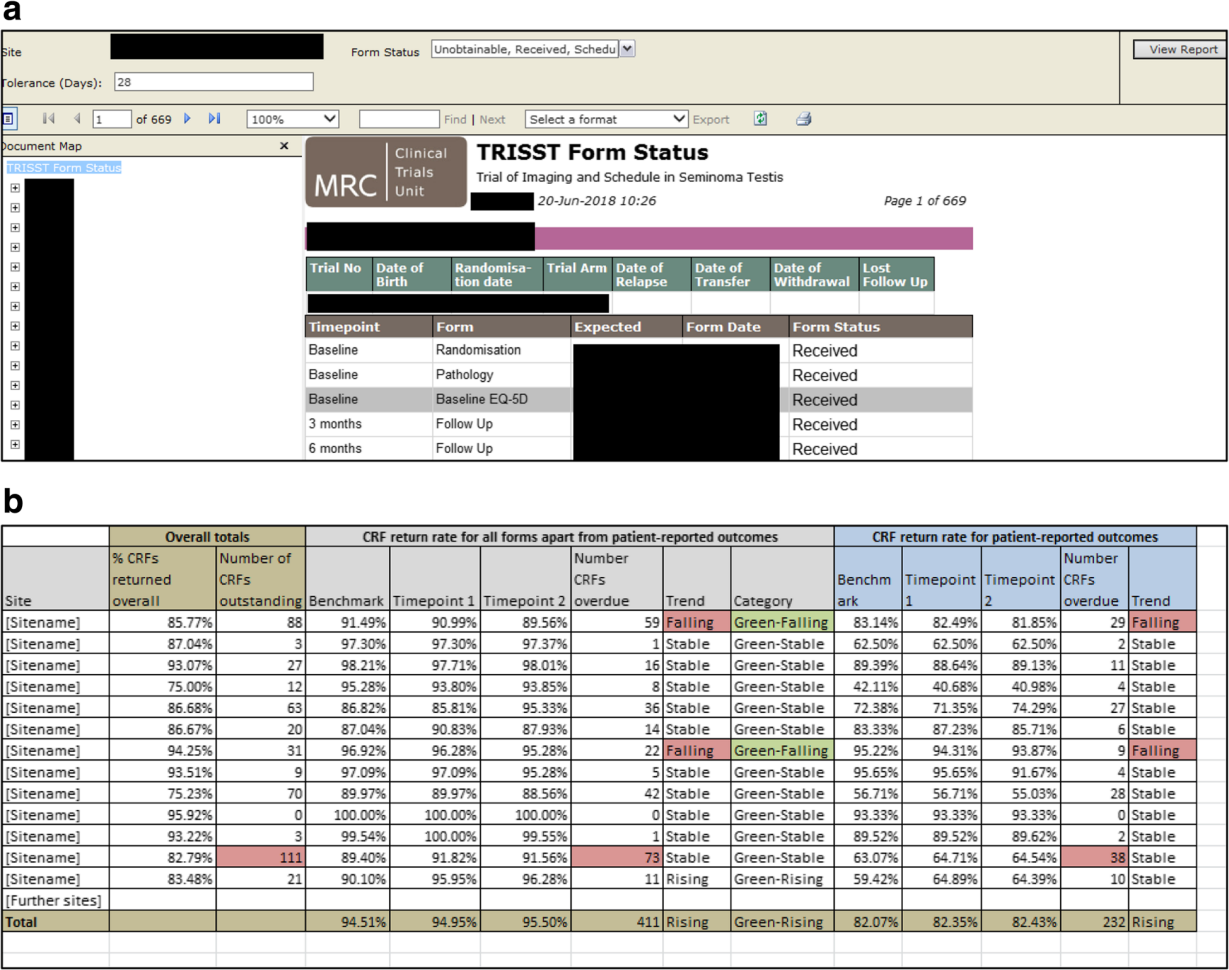
**a** Screenshot of automated form status report. **b** Screenshot of Excel-based data return rate trend tool. CRF Case Report Form, MRC Medical Research Council, TRISST Trial of Imaging and Schedule in Seminoma Testis

Figure 3 shows a summary of the systems and centralised methods employed to monitor and manage the DRR as part of the new processes in TRISST. Our first aim was to visualise change over time in each centre’s DRR. Automated, validated reports, developed in house, were already in use for reviewing the current DRR overall and per centre, and listing the status of each form (see Table 1 for possible statuses). We developed an Excel-based tool to store report extracts from different points in time, allowing review of per-centre change in the DRR (see Fig. 4). We used Excel because our in-house reporting system was not designed to store data extracts over time, and because Excel was deemed sufficiently robust, and user friendly, for the task. The tool underwent testing prior to use to check that the calculations were correct for each centre. We aimed to automate the tool as much as possible, without the need for any manual data manipulation or formula adjustment. A new batch of data can be added into the tool in only a few short steps, amounting only to copying the data into a blank worksheet and instructing formulas to look at the new data. Clear and concise instructions were presented within the tool to minimise the risk of copy-and-paste errors.

We added new data extracts before each formal CTU team meeting (about every 6–8 weeks). After around 2 months of collecting these data, we were able to see trends across at least two timepoints against an initial ‘benchmark’. Centres were no longer simply categorised as green for acceptable (≥80% DRR) and red for unacceptable (< 80%), but had one of four categories with an incorporated time component:

- green-stable or green-rising (≥80% data returns and either no trend or rising);
- green-falling (≥80% data returns but consistently falling over two timepoints);
- red-rising (< 80% data returns but consistently rising over two timepoints); and
- red-falling or red-stable (< 80% data returns and falling or no trend).

Rises or falls could be of any magnitude to count towards a trend (but the use of two timepoints means that short-term issues are discounted). The centre trend data were summarised and reviewed in the team meetings to help decide which centres required intervention regarding DRR.

In order to better understand the problems at a given centre, for DRR reporting, both within the team and externally (e.g. to the TMG), we started to show patient-reported outcome forms separately, as well as showing all forms in totality. While (in this setting at least) overdue staff-completed forms can usually be returned at any time, patient-completed forms cannot be completed retrospectively, and may be justifiably missing if a patient has chosen not to complete. If centres confirm that a patient-reported outcome form was not completed at the expected time, it is categorised as ‘unobtainable’ (i.e. permanently missing). A centre with low data returns solely because of missed patient-completed forms may have protocol compliance issues rather than just data return issues. Alternatively, this could indicate that the protocol’s data collection processes are not feasible. In addition, if the unobtainable patient-completed forms are not discounted from the overall DRR, some centres may end up with permanently low scores for the remainder of the trial, regardless of subsequent data return performance. This does not help the trial team distinguish current from historical issues, and may be demotivating for participating centres. For an example, see the fourth centre in Fig. 4b. This has a low number of CRFs currently outstanding (12 CRFs) but a low overall DRR (75%) because of issues with patient-reported outcome return earlier in the trial, now resolved through discussion with the principal investigator.

### Initial management of highlighted centres

The categorisation described allowed more nuanced approaches to communications with centres. As we had done previously, we notified centres with DRR < 80%, but, if they were showing improvement (i.e. ‘red-rising’), we could now acknowledge this in our communication. We also began to contact centres with ≥ 80% data returns but consistently falling (i.e. ‘green-falling’), to notify them that they did not seem to have sent us any CRFs for a while. Centres with no apparent issues (i.e. ‘green-stable’ or ‘green-rising’ in the category list) were not contacted specifically about data return.

Based on previous lack of success at some centres in simply asking for all outstanding data, we modified our approach to dealing with the ‘red’ centres. We agreed that, at most times, there was no particular reason why we would need all overdue data immediately. We agreed that it would therefore be sufficient (and more feasible) for problem centres to send data at a higher rate than new CRFs were becoming due from the occurrence of patient visits. We also hypothesised that this approach might be better received by centres than requests for all data immediately. We approached centres with a proposed portion of the overdue forms to send within a certain timeframe (e.g. 20 forms within 2 weeks). Where possible, we tried to make an agreement in writing with centres about this rather than dictate, and there was sometimes some room for negotiation, so long as this ‘repayment plan’ would result in an improved DRR over time. After the agreed timeframe, we would review the centre’s DRR, discuss with them again and agree another target if the DRR was still below the acceptable threshold.

### Escalation policy

We formalised an escalation policy, based on previous trial processes, to deal with centres that either did not respond to initial contact or, in the view of the CTU team, had consistently failed to meet the targets they had agreed to. For prolonged issues, the CTU team would involve the centre’s principal investigator and other relevant individuals, such as network managers, in discussions. At the highest level, we planned to escalate to internal quality management teams within the CTU, agree a potential action with the TMG and consider an on-site visit with a focus on improving the data returns (rather than carrying out any other monitoring activities). At each stage of the policy, we agreed we would try to ‘de-escalate’ where possible, through discussion with the centre. It was designed around the importance of a collaborative approach, with opportunity at all stages for agreed, rather than dictated, deadlines. We aimed to discuss issues with centres to understand the reasons for low data returns, as part of the collaboration.

### Evaluation

We conducted a post-hoc, descriptive analysis of the effectiveness of the new processes.

## Results

The new processes were implemented in the trial in April 2015, and stopped in September 2016 due to staffing changes on the trial. During this time, the per-month expected number of CRFs was near its peak for the trial (see Fig. 2c).

The DRR had generally been high during the trial (Fig. 2a), but peaked in May 2016 at nearly 95% (the highest figure in any TMG report before or since). The number of centres beneath the acceptability threshold, at a median of 6 in the trial until the end of 2014, fell to figures of 3, 1 and then 0 in subsequent meetings (Fig. 2b). There had never previously been a time when all centres were above the threshold. In the month of this TMG report (May 2016), the expected number of CRFs arriving was still above the median for any month across the trial.

**Fig. 5:**
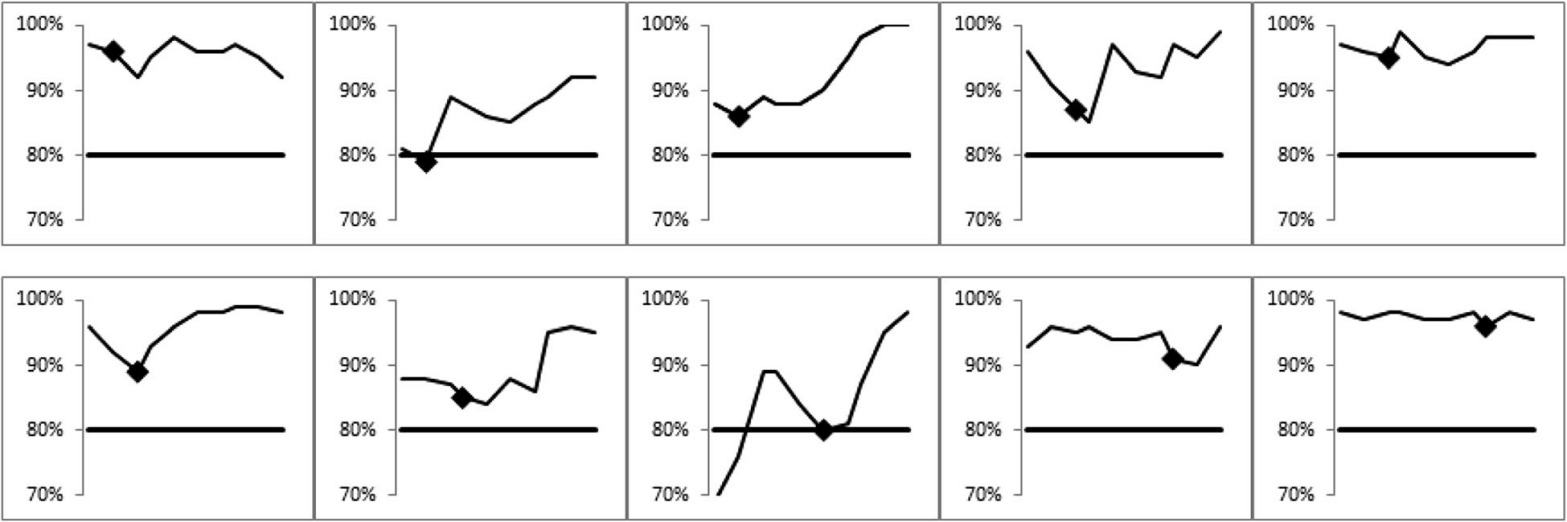
Data return rates of centres contacted regarding falling data return rates between 13 May 2015 and 28 September 2016. Thick black line in each plot indicates the 80% acceptability threshold. Marker on each line is the date of the team meeting at which it was agreed to contact the centre

Over the course of using the new process, there were 10 CTU team meetings at which DRR figures were reviewed. Across these, 10 centres were highlighted for action based on having acceptable but dropping rates. These centres were contacted, usually to simply notify them that they appeared not to have sent us data recently. Apart from discussion arising from this initial contact, these centres were not contacted again prior to subsequent CTU team meeting review. Figure 5 shows changes in these centres’ rates after being contacted. In 6/10 centres, we observed a rise in data returns by the time of the next CTU team meeting. In the remainder, there was a rise at the second CTU team meeting after notification. During this time, we continued to look for and act upon other data return issues, such as centres with consistently low rates, or centres with a relatively large number of CRFs outstanding, whatever the return rate.

We successfully implemented the new collaborative approach to dealing with centres with larger backlogs, as shown by all centres being within the acceptability threshold by May 2016. One centre in particular with historical data return issues (median of 65% in TMG reports from the start of the trial to the end of 2014) was brought up as high as 90% in late 2015. We also successfully implemented the separation of forms that could and could not be completed retrospectively, and this helped inform our understanding of each centre’s specific issues.

At no stage did we decide we needed to use the prepared escalation policy. Some centres had problems, but all were receptive to our approach of reaching consensus with them on a suitable action.

## Discussion

We present here preliminary effectiveness evidence for novel methods to monitor and manage the DRR in trials, an area that has so far received very little attention. The addition of a time dimension to our reports allowed us to see downward trends before they became problems, and added nuance to our handling of known problems. Early contact with centres noted to have falling return rates elicited an improvement in rates usually within 6–8 weeks. Availability of separate data for forms that cannot be completed retrospectively, as well as overall figures, allowed us to better understand what problems were occurring at individual centres, and to further tailor our approach accordingly.

Working with centres to resolve problems collaboratively resulted in all centres’ DRR lying within the trial’s acceptability threshold, which had not happened before. Although we designed a comprehensive escalation policy to handle persistent problems, we did not have to resort to this. There was a sense among the CTU team of increased oversight of data returns, particularly due to the temporal data we were now reviewing. Production of the additional report was not time consuming or difficult once the processes had been put in place. The new processes were developed and implemented without any additional trial manager, data manager or programmer resource. They were stopped after an initial period, not because they were burdensome, but because new staff on the trial had different preferences for how to handle this aspect of trial management. Now, after completing this post-hoc evaluation of the methods, we may look to implement them more widely within our trials units, possibly in a more automated fashion.

In general, we recommend these methods to be used flexibly and pragmatically. We suggest the reporting methods are used to highlight possible problem centres, but that action is decided through CTU team or TMG discussion. For example, centres might not be contacted immediately about corrective actions if they have already notified the CTU team that they currently have temporary resourcing issues, or if the absolute number of overdue forms is very low. In that case, the CTU team should instead arrange with the centre a point in the near future to discuss again to see whether things have improved. The optimal acceptability thresholds may vary between trials due to factors including the number of CRFs, the duration of the trial, the size of each participating centre and the trial’s characteristics (e.g. phase, presence of an Investigational Medicinal Product, etc.). They may also vary within trials, or between CRFs of different types (e.g. adverse event data may be treated differently to other data).

Although the described reporting methods were simple to use once set up, there were some associated challenges. Development of this particular system first required detailed, validated database reports; these were based on a family of similar reports developed for trials at the MRC CTU at UCL, but these may not be available in other institutions. Secondly, we required a good knowledge of Excel to turn the report extracts into DRR trend data. Resolving any spreadsheet problems that arise later may be difficult if users at the time are not familiar with Excel formulas. We do not consider this a significant barrier, as use of the spreadsheet once set up is simple, and a similar result could be achieved by a statistician using statistical software. We have not yet, however, incorporated the trend data into an automated report. This would require storing of data from each report snapshot within the reporting platform; it currently cannot do this. Such a solution would offer better usability for the CTU team (especially for those less familiar with Excel), but would not make much difference to the result.

Although producing and reviewing the DRR figures was relatively simple, liaising with centres to resolve issues could sometimes be time consuming (e.g. making initial contact, following up with phone calls, checking on progress, etc.). While this may be somewhat more effort than simple reminders for centres with unacceptable DRR, we consider the time worth spending if it achieves high data returns which, in turn, support other, important trial processes. The possible effect of good negotiation and communication skills is harder to quantify, but it seems likely that centres respond better when they are involved in discussion about how to improve data returns, rather than issued with demands for data. Training in negotiation, communication and influencing skills may be a useful part of general training for the trial manager and data manager roles.

We sometimes found difficulties in reporting detailed DRR figures to others, for example the TMG, as they were used to simpler, less nuanced methods. However, we believe this is surmountable given time and familiarity with the new methods; in any case, the more detailed statistics may be more useful in day-to-day trial management than for reporting to oversight committees.

There are several caveats to present in the interpretation of our results. At the time of implementing our methods, TRISST had finished recruitment, and the main focus both at centres and at the CTU was therefore data collection. This contrasts to earlier in the trial, when centres and the CTU were focused on recruitment, and the CTU also on expanding and promoting the trial. It is also true that this process was implemented at a time when the expected per-month number of CRFs arriving was declining; nonetheless, the months in which we used the new methods were among the busiest in the trial in terms of CRFs expected.

As we did not have cause to use the escalation plan, we cannot be sure whether this works for centres with persistent problems. It is not obvious what leverage we would have to encourage continued data returns. Unlike recruitment, for which centres are formally accountable to Clinical Research Networks in the UK [24], there are—aside from basic requirements of adherence to Good Clinical Practice, the UK Policy Framework for Health and Social Care Research and other standards—no significant incentives to help ensure ongoing completeness of follow-up data. Trialists, however, also have a responsibility to ensure that the amount and type of data being requested is justified and reasonable. It is recognised that non-priority data items can make up a large proportion of all follow-up data requested [25, 26]. This may conflict with data protection principles which dictate that personal data should be adequate, relevant and limited to what is necessary for their explicitly stated purposes, particularly in the light of strengthened data protection legislation in the European Union [27]. Incentives for centres to provide follow-up data would increase the onus on trialists to justify the amount of follow-up data they request.

TRISST collects data on paper CRFs, and an increasing number of trialists are adopting electronic data capture [8]. However, we believe our methods apply equally to these trials, as data completion still needs to be monitored and issues managed efficiently.

We recognise that the methods described may not produce quick results, and therefore may not be suitable in short-duration trials.

Our methods do not deal with all data completeness issues, and additional processes are required to address other aspects of data quality and integrity. The methods help us collect CRFs from centres, but additional action is clearly required if data on received CRFs are missing. It is also necessary to look at exactly which forms are missing for a given centre. For example, a return rate of 90% is good, but on closer inspection you may find that the 10% of overdue forms are all important data that was due several months ago. You could detect this by additional, complementary methods, such as listing all CRFs more than 6 months overdue. It is beyond the scope of this work to explore the effects of our methods on other aspects of data quality (e.g. accuracy of data provided), but this could be included in future work in this area.

Our methods mainly address expected, scheduled forms. Additional processes are required to ensure unscheduled forms (e.g. to record serious adverse events or deaths) are reported in a timely manner [28], especially as these often contain information that needs to be reported urgently. Methods for identifying missing unscheduled forms might include: specific CRF questions to help ascertain whether an unscheduled CRF might be required; use of electronic health record data to look for unreported events of interest (e.g. deaths or serious adverse events); or comparing the number of unscheduled CRFs received across participating centres or against an expected minimum threshold [29].

Many trials have more participating centres than TRISST (35 centres), and are more demanding in terms of follow-up data. It remains to be seen what the resource implications are for scaling up these methods into a larger study, particularly during the recruitment phase, or into studies with greater safety reporting requirements.

The described methods rely on CTU data entry of paper forms being up to date. However, as we consider this good practice, this should not be a limitation per se. Data completeness may well be impacted by the way centres are organised and resourced, but it is beyond the scope of this article to explore such factors.

## Conclusions

Preliminary evidence suggests that central monitoring of the DRR using statistics to show changes over time, and managing issues through a nuanced, collaborative approach, can result in a high DRR overall and across all centres. This is an important issue with very limited evidence to support best practice. The evidence we present here is also limited, but the methods we propose could be tested in a more robust fashion at very little cost or risk (e.g. as a study within a trial [30]). If they are proven to be effective, these methods could benefit participating centres, CTUs, sponsors and even trial participants through increased efficiency and enhanced clinical trial oversight.

## Abbreviations

CRF: Case Report Form
CTU: clinical trials unit
DRR: data return rate
MRC CTU at UCL: Medical Research Council Clinical Trials Unit at University College London
TMG: Trial Management Group
TRISST: Trial of Imaging and Schedule in Seminoma Testis
